# WHY DON’T OLDER ADULTS USE SENIOR CENTERS? EVIDENCE FROM A SAMPLE OF MASSACHUSETTS ADULTS AGE 50 AND OLDER

**DOI:** 10.1093/geroni/igz038.1845

**Published:** 2019-11-08

**Authors:** Ceara Somerville, Nidya Velasco Roldan, Cindy N Bui, Caitlin E Coyle

**Affiliations:** 1 University of Massachusetts Boston, Boston, Massachusetts, United States; 2 Gerontology Institute, University of Massachusetts Boston, Boston, Massachusetts, United States

## Abstract

Senior centers are an integral community resource, providing programs and services intended to meet the vast range of needs and interests of older adults. There is a growing literature describing senior center participants and benefits to participation, but little is known about those who choose not to participate at a local senior center. This presentation uniquely characterizes non-users of senior centers, based on a sample of community-dwelling adults aged 50+ from seven communities in Massachusetts (N = 9,462). To date, this is the largest data set that describes senior center usage. Most of the sample were women (60%) and in the 60-69 age group (36%). More than three quarters of the sample do not use the local senior center (77%). The most common reasons for non-usage were lack of interest (27%) and not feeling old enough (26%). There are significant differences in reasons of non-usage among age groups and gender (p < .001). Younger age groups’ (50-69) most popular reasons for non-usage were not feeling old enough, not having time, inconvenient senior center hours, and not knowing what is offered. In contrast, older age groups (80+) more frequently reported having no interest or using programs elsewhere. Men were more likely to report not being interested and not being familiar with what is offered. Women were more likely to report not having time, inconvenient hours of programming, and using programs elsewhere. Based on results from this study, this presentation will outline implications for the future of senior centers and their programming.

